# Appropriate trust in online health information is associated with information platform, commercial status, and misinformation in patients with high cardiovascular risk

**DOI:** 10.1177/20552076251334438

**Published:** 2025-04-29

**Authors:** Woei Xian Lim, Hooi Min Lim, Yew Kong Lee, Carmen Jia Wen Chuah, Adina Abdullah, Chirk Jenn Ng, Adam G Dunn

**Affiliations:** 1 Department of Primary Care Medicine, Faculty of Medicine, 37447 Universiti Malaya, Kuala Lumpur, Malaysia; 2 Department of Research, 68752 SingHealth Polyclinics, Singapore; 3 121579 DUKE-NUS Medical School, Singapore; 4 Biomedical Informatics and Digital Health, School of Medical Sciences, 522555 Faculty of Medicine and Health, The University of Sydney, Sydney, Australia

**Keywords:** Online health information, trust, quality, cardiovascular, misinformation

## Abstract

**Background:**

The quality of online health information (OHI) on cardiovascular health is highly variable. Trusting poor quality OHI can lead to poorer health decisions. This study examined information characteristics associated with appropriate trust in OHI among patients with high cardiovascular risk.

**Methods:**

This is a secondary analysis from a cohort study of 270 participants with high cardiovascular risk from a primary care clinic in Malaysia. Participants recorded OHI entries and their trust levels over 2 months using a digital diary. Overall, 1194 OHI entries were included and categorised by platform, commercial status, content focus, and presence of misinformation, and assessed for quality using the DISCERN tool. Appropriate trust was determined by trust-quality matching (trusting high quality or distrusting low quality OHI). The association between information characteristics and appropriate trust was analysed using multiple logistic regression.

**Results:**

Most entries were from websites (62%) and non-commercial sources (88.2%). Misinformation was found in 23.3% (278 of 1194) of entries; 30.8% (367 of 1194) were of good or excellent quality; 51.5% (615 of 1194) were appropriately trusted. Information from websites (vs social media) (AOR 4.31, 95% CI 3.14–5.91, *P* < .001), non-commercial source (vs commercial) (AOR 1.59, 95% CI 1.01–2.50, *P* = .047), and absence of misinformation (vs presence of misinformation) (AOR 2.11, 95% CI 1.40–3.20, *P* < .001) were associated with higher appropriate trust.

**Conclusions:**

OHI from websites, non-commercial sources, and information without misinformation has higher appropriate trust among patients with high cardiovascular risk. This study highlighted the need for good-quality OHI and dissemination through reliable sources.

## Introduction

There is an increased use of online health information to inform health decision-making among the public,^1,2^ but the quality of online health information is highly variable and misinformation is common.^3,4^ Online health information related to cardiovascular diseases can be misleading, or promote alternative treatments for which there is limited or no evidence of safety and effectiveness. For example, drinking an inappropriate amount of water to treat hypertension and the use of statins have been linked to memory loss, cataracts, pancreatic dysfunction, and cancer.^5–7^ Consequently, patients may trust poor quality online health information, resulting in adverse consequences such as non-adherence to prescribed treatments and delay in seeking medical attention from healthcare professionals.^8,9^

Trust in online health information is an important factor that influences patients’ health decisions. Internet users often base their trust on their perception of the quality of information.^10^ The source and content of the information directly influence this trust and affect how they use the information to make health decisions.^11^ However, many individuals lack the necessary skills to appraise the online health information they encounter;^12,13^ they often do not objectively assess the credibility of online health information and may trust poor quality online health information.^14,15^

The quality of online health information varies based on several characteristics, including the platform, content, commercial status and presence of misinformation.^16,17^ Platform refers to the channel that delivers the information, such as websites or social media. Websites generally provide higher-quality information, while social media tends to have poorer quality due to the lack of regulations governing veracity.^18,19^ Content refers to the focus of the information, with information on general wellness exhibiting lower proportions of good quality outcomes as compared to those focused on specific diseases or conditions.^20,21^ Commercial status refers to the presence of financial backing behind the information, such as through advertisements or sponsorships.^16^ Commercially sponsored content is less trusted compared to non-commercial information, perceiving it as biased.^2,22^ The presence of misinformation can reduce trust, as individuals tend to be more sceptical and doubtful about the accuracy of sources that contain false or misleading claims.^23^

While online health information is widely available, prior research has often relied on pre-prepared vignettes or hypothetical scenarios, which may not fully capture real-life information-seeking behaviours.^24,25^ It remains unclear how online health information characteristics influence trust, and whether patients can appropriately trust online health information (trusting good-quality information and distrusting poor-quality information). This study addresses this gap by analysing real-life online health information encounters among patients with high cardiovascular risk, offering more contextually relevant insights. This study aimed to examine the association between online health information characteristics and appropriate trust in online health information.

## Methods

### Study design

This is a quantitative study using secondary analysis from the data of a cohort study, which aimed to examine the online health information-seeking behaviour in participants with high cardiovascular risk.^26^ A total of 270 participants were recruited in the original cohort using a universal sampling method between October 2022 and January 2023 in a primary care clinic at the University of Malaya Medical Center (UMMC), Kuala Lumpur, Malaysia. Participants who met the following inclusion criteria were included in the original cohort study: participants (a) aged 18 years or older; (b) have high cardiovascular risk where statin use was indicated as by Malaysian dyslipidaemia management guidelines;^27^ and (c) regularly use a smartphone.

Participants were asked to use a digital information diary tool (Supplementary material 2) to record the health information that they encountered for 2 months. For each entry, they first selected the source of information, then chose a relevant sub-category, and finally rated their trust levels of each information using a 5-point Likert scale, where 1 indicated the lowest level of trust and 5 indicated the highest. A more detailed explanation of the information diary tool can be found in our previous publication.^26^

### Conceptual framework

**Figure 1.**
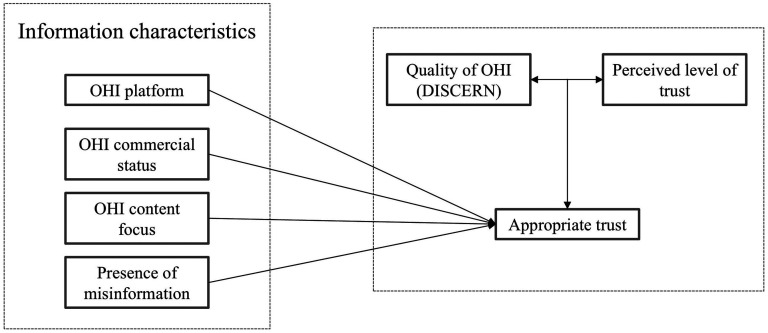
The conceptual framework shows that appropriate trust is determined by comparing the quality of online health information (OHI) with the perceived level of trust and determines the association between four information characteristics (platform, commercial status, content focus and presence of misinformation) and appropriate trust.

A conceptual framework (Figure 1) to study appropriate trust and online health information quality was developed based on the literature review and Kelton et al.'s integrated model of trust in information. In Kelton's model, the source and trustworthiness of information act as the key determinants of trust in information.^11^ Four main factors influence information trustworthiness: accuracy, objectivity, validity, and stability, all of which are related to the content of information.^11^ Information trustworthiness directly influences an individual's trust, though other factors – such as prior knowledge, recommendations, and the relevance of information – can also play a role.^11^ However, trust in online health information develops in stages. Sillence et al.^28^ proposed a staged model of trust, where individuals initially rely on heuristic cues ­– such as website design and source credibility – for quick judgements. A deeper evaluation occurs when users find the information personally relevant and have the ability, such as prior knowledge, to analyse its quality in detail**.** However, since deep analysis requires time and capacity, users selectively choose which information to analyse thoroughly and which to judge based on heuristic cues.

Our study model examines the relationship between the actual quality of online health information and patients’ perceived trust in that information. The actual quality was measured using the DISCERN score rated by the researchers, while perceived trust was based on patients’ ratings in the diary tool. By comparing these two factors, we determined whether trust was appropriate (‘Appropriate trust’), meaning patients appropriately trusted high-quality information or appropriately distrusted low-quality information. As Kelton's model emphasised the role of information source as a precursor for trust, we further examined whether four key information characteristics ­– platform, commercial status, content focus, and presence of misinformation – were associated with appropriate trust. This framework allows us to assess not only whether patients’ trust aligns with actual quality but also how these information characteristics are associated with appropriate trust.

### Inclusion and exclusion criteria of online health information entries

To be included in the analysis, online health information entries had to be accessible (e.g., valid links or images with the source visible) and written in English, Chinese or Malay. Online health information entries were excluded if they were too short or had no description, were duplicated from the same participant, were unrelated to health, were not from online sources (e.g. newspaper, magazine, booklet, pamphlet, or infographics), or only showed search results pages on search engines without specifying the visited website.

### Data extraction

We screened each online health information entry using the inclusion and exclusion criteria. As each entry only included the link or screenshot, we accessed each online health information source individually to check if the source was active and included it in the analysis if it was active at the time of checking. For URL links, we assessed and analysed the information directly from the source to which the links were directed. In cases involving screenshots, images, or descriptions with incomplete content, we conducted online searches to identify the sources of the information.

Entries were categorised based on information characteristics, which focus solely on their content: information platform (website or social media), commercial status (non-commercial or commercial), content focus (condition-related or general well-being topics), and presence of misinformation (yes or no).

To determine the quality of information, two researchers with medical qualifications (WXL, CJWC) independently rated each entry using the DISCERN tool, which consists of 16 questions to assess information reliability, treatment options and overall quality based on a 5-point Likert scale.^29^ Scores obtained from the tool were categorised into five categories: Very poor (scores between 15 and 26), Poor (scores between 27 and 38), Fair (scores between 39 and 50), Good (scores between 51 and 62), and Excellent (scores between 63 and 75).^30^ Researchers met at intervals of 50 entries to compare and discuss the results. When there was any discrepancy between the categories, a consensus was sought from a third researcher (HML). The quality of each information was compared with the trust level rated by participants. The trust was categorised as appropriate or inappropriate.

To identify the presence of misinformation, both researchers (WXL, CJWC) independently assessed the content, using Wardle's misinformation taxonomy as a guide.^31^ This taxonomy encompasses misinformation categories such as satire, misleading content, imposter content, fabricated content, false connection, false content, and manipulated content. Both researchers (WXL, CJWC) determined if information falling under any of the taxonomy categories was classified as misinformation. When there was any discrepancy in determining the presence of misinformation, a consensus was sought from a third researcher (HML). Inter-rater reliability, measured by Cohen's kappa (κ) value, showed a pre-discussion agreement of 0.919 for classifying misinformation.

Appropriate trust (appropriate vs inappropriate) was determined by comparing the level of trust rated by participants and the quality of each online health information entry (Table 1). Being unsure about good quality or poor quality information was classified as inappropriate. This is because good quality information merits stronger trust as it provides reliable information to help make better-informed health decisions, whereas poor quality information often contains misinformation or inaccurate information, potentially leading to harmful health decisions. For fair-quality information, both trusting and not trusting were considered appropriate, as fair-quality content is neither explicitly high quality nor entirely misleading. Given this ambiguity, individuals may reasonably choose to trust or not trust, making both responses appropriate.

**Table 1.** Matrix of trust-quality matching to determine the appropriate trust in online health information quality.

Quality / Trust level	Completely trust	Trust	Unsure	Not trust	Do not trust at all
Excellent	Appropriate	Appropriate	Inappropriate	Inappropriate	Inappropriate
Good	Appropriate	Appropriate	Inappropriate	Inappropriate	Inappropriate
Fair	Inappropriate	Appropriate	Appropriate	Appropriate	Inappropriate
Poor	Inappropriate	Inappropriate	Inappropriate	Appropriate	Appropriate
Very poor	Inappropriate	Inappropriate	Inappropriate	Appropriate	Appropriate

### Data analysis

Data were analysed using the Statistical Package for Social Sciences (SPSS) version 20 for descriptive and inferential analyses. A descriptive analysis was conducted to report on the numbers and percentages of participants’ sociodemographic profiles (age, gender, ethnicity, language, education level, household income per month), the presence of CVD (yes/no), and eHealth literacy scale (eHEALS).^32^ The eHEALS measures a total of 8 items including overall knowledge, confidence, and perceived ability to locate, evaluate, and use electronic health information for health-related decisions.^32^ The total eHEALS score ranges between 8 and 40 with a higher score indicating higher eHealth literacy. It has been validated in Malaysia, and the internal consistency was assessed using Cronbach's alpha, which was .883.^15^ A descriptive analysis of the online health information entries was reported based on platform, commercial status, content focus, and presence of misinformation. Dependent variables were described using the DISCERN categories of the online health information quality (Excellent, Good, Fair, Poor, Very Poor), participants’ levels of trust (Completely trust, Trust, Unsure, Not trust, Do not trust at all), and Appropriate trust (Appropriate, Inappropriate). The distribution of level of trust and quality comparing the subcategories of four information characteristics (website vs social media; commercial vs non-commercial; condition-related vs general well-being; misinformation vs no misinformation) was reported using descriptive analysis.

Inferential analysis was conducted to determine information characteristics associated with appropriate trust. This was done using multiple logistic regression between appropriate trust categories as the dependent variable and the characteristics of information as independent variables. If *P* < .05, the result is considered significant.

## Results

**Figure 2.**
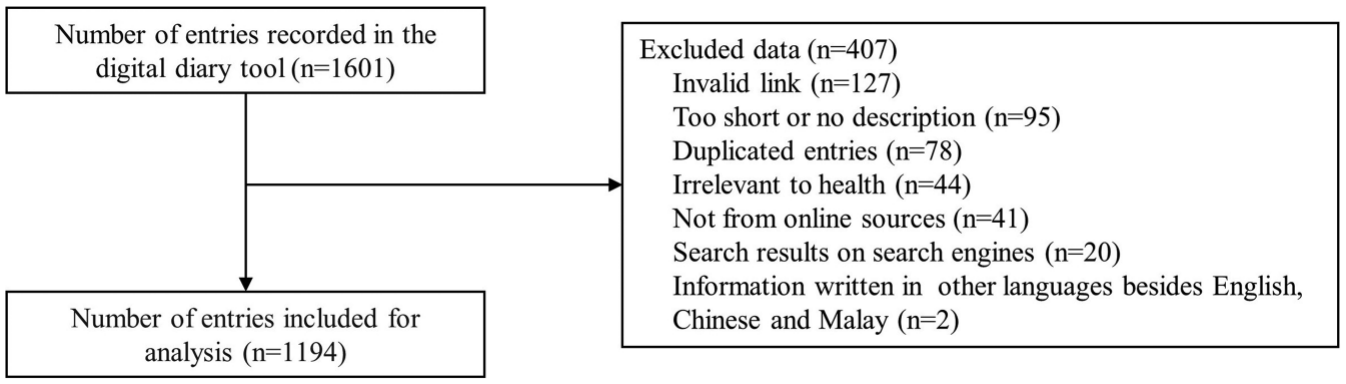
Flowchart of entries included in this study.

A total of 1194 of 1601 entries from 128 participants were included in the analysis (Figure 2). Overall, 407 entries were excluded, with the majority being due to an invalid link (*n* = 127). The number of included entries per patient ranged from 1 to 97; the median was 3.5 (IQR: 2–12).

### Sociodemographic characteristics of study participants

The mean age of the study population was 60.2 ± 10.05 years. Most participants (69.5%, 89 of 128) had a pre-university or tertiary education qualification (Table 2). A history of cardiovascular disease was reported for 25% (32 of 128) participants. The mean eHealth literacy score of participants was 29.2 ± 4.79.

**Table 2.** Sociodemographic characteristics of participants (*n* = 128).

Characteristic		*n*	%	Mean ± SD
Age(years)				60.2 ± 10.05
Gender	Male	65	50.8	
	Female	63	49.2	
Ethnicity	Malay	53	41.4	
	Chinese	58	45.3	
	Indian	15	11.7	
	Others	12	1.6	
Educational level	No formal education/lower secondary school	7	5.5	
	Upper secondary school	32	25	
	Pre-university	32	25	
	Tertiary education	57	44.5	
Monthly household income	<RM 3000	49	38.3	
	RM 3000-6000	44	34.4	
	> RM 6000	35	27.3	
Presence of cardiovascular disease	Yes	32	25	
	No	96	75	
eHealth literacy score				29.2 ±4.79 (range: 14-40)

### Information characteristics

For information platforms, websites served as the primary platform, comprising 62% (*n* = 740) (Table 3). Within social media, Facebook is the most used, with 20% (*n* = 239), followed by YouTube at 15.2% (*n* = 181). Examining the commercial status, non-commercial entries dominated at 88.2% (*n* = 1053). Among the non-commercial entries, most of the online health information was from news and media (*n* = 388, 32.5%), followed by government and organisation (*n* = 176, 14.7%). Regarding the content focus, entries were evenly distributed between general well-being (*n* = 612, 51.3) and condition-related topics (*n* = 582, 48.7%). Participants looked at information related to the disease (*n* = 385, 32.2%) the most, followed by complementary and alternative medicine (*n* = 385, 32.2%). Most entries did not contain misinformation (*n* = 916, 76.7%).

**Table 3.** Information characteristics of the online health information entries (*n* = 1194).

Characteristic	Variable	*n*	%
Platform	Website	740	62.0
	Social media	454	38.0
Commercial status	Non-commercial	1053	88.2
	Commercial	141	11.8
Content focus	General well-being	612	51.3
	Condition-related	582	48.7
Presence of misinformation	No	916	76.7
	Yes	278	23.3

### Level of trust and quality of online health information

The breakdown of online health information quality reveals that 30.8% (367 of 1194) of entries were classified as Good or Excellent, while 37.1% (443 of 1194) fell into the Poor or Very Poor category (Table 4). 64% (764 of 1194) of participants indicated Trust or Complete trust in the online health information, while 34.4% (411 of 1194) were Unsure whether or not to trust. When analysing the relationship between online health information quality and perceived trust levels, 51.5% (615 of 1194) of entries were trusted appropriately.

**Table 4.** Quality, level of trust and appropriate trust in online health information (*n* = 1194).

Variable		*n*	%
Quality	Very poor	148	12.4
	Poor	295	24.7
	Fair	384	32.2
	Good	329	27.6
	Excellent	38	3.2
Level of trust	Do not trust at all	13	1.1
	Not trust	6	0.5
	Unsure	411	34.4
	Trust	376	31.5
	Completely trust	388	32.5
Appropriate trust	Appropriate	615	51.5
	Inappropriate	579	48.5

### Distribution of online health information quality and level of trust relative to four information characteristics

Information from websites had better quality and higher trust than social media platforms (Supplementary 1). Information from social media is of poorer quality (71.1%, 323 of 454). Information with non-commercial intent had better quality and trust than commercial information. Information from commercial sources has poorer quality (68.8%, 97 of 141). For content focus, condition-related topics tended to be of better quality and higher trust than general well-being. Trust in social media is high (46.3%, 210 of 454) despite poor quality. Trust in general well-being information is also high (52%, 287 of 612) despite poor quality (51.8%, 317 of 612). For entries with misinformation, 60.8% (169 of 278) were rated as unsure of trust.

### Information characteristics associated with appropriate trust

In multivariate logistic regression, information from websites had a higher appropriate trust than information from social media (B = 1.46, SE = 0.16, Wald = 82.01, AOR 4.31, 95% CI 3.14–5.91, *P* < .001) (Table 5). Non-commercial information had higher appropriate trust compared to commercial information (B = 0.46, SE = 0.23, Wald = 3.95, AOR 1.59, 95% CI 1.01–2.50, *P* = .047). Information without misinformation had a higher appropriate trust than those with misinformation (B = 0.75, SE = 0.21, Wald = 12.53, AOR 2.11, 95% CI 1.40–3.20, *P* < .001).

**Table 5.** Univariable and multivariable logistic regression of four information characteristics associated with appropriate trust in online health information.

Characteristic	Variable	Appropriate trust (%)	Unadjusted OR (95% CI)	*P*-value	Adjusted OR (95% CI)	*P*-value
Platform	Website Social media	450/740 (60.8) 91/454 (20.0)	6.19 (4.71–8.13) 1	<.001	4.31 (3.14–5.91) 1	<.001
Commercial status	Non-commercial Commercial	504/1053 (47.9) 37/141 (26.2)	2.58 (1.74–3.83) 1	<.001	1.59 (1.01–2.50) 1	.047
Content focus	Condition-related General well-being	320/582 (55.0) 221/612 (36.1)	2.16 (1.71–2.73) 1	<.001	1.11 (0.84–1.45) 1	.467
Presence of misinformation	No Yes	494/916 (53.9) 47/278 (16.9)	5.75 (4.10–8.08) 1	<.001	2.11 (1.40–3.20) 1	<.001

Adjusted for: platform, commercial status, content focus and presence of misinformation.

## Discussion

### Summary

Our results showed that the overall quality of online health information encountered by patients with high cardiovascular risk was poor. Participants demonstrated appropriate trust in 51.5% of online health information entries. They were more likely to appropriately trust information from websites compared to social media, non-commercial sources compared to commercial ones, and information without misinformation compared to information containing misinformation.

### Comparison with relevant literature

Our study showed that participants with high cardiovascular risk prefer to seek online health information from websites rather than social media. These findings align with other previous studies conducted among patients with chronic diseases such as cancer and inflammatory bowel disease.^33,34^ Examining the commercial status, our study revealed a predominance of non-commercial information (88.2%). This is similar to a study conducted in arthritis patients (80.8%),^35^ but contrasted with findings from a study conducted among the general public in the US, which documented a higher prevalence of commercial websites (71.8%).^36^ Discrepancies in prevalence may stem from various factors, such as age and health literacy as older adults prefer health information from academic-affiliated sources, and people with high health literacy prefer information from healthcare professionals.^37,38^

The quality of online health information in our study exhibited variability, with 37.1% falling into the Poor or Very Poor categories, followed by 32.2% in the Fair category, and 30.8% in the Good or Excellent categories. This finding is aligned with other previous systematic reviews, highlighting a high variability in online health information quality.^39,40^ The results showed that the information encountered on websites was higher quality than on social media, aligning with two other studies that examined the quality of information on COVID-19 and surgical procedure using the DISCERN tool across websites and social media.^22,41^ The non-commercial information that participants encountered was higher quality compared to the commercial information they encountered. This finding is aligned with studies which used the DISCERN tool to evaluate surgical and chronic disease information,^42,43^ but one study focusing on joint replacement information showed higher quality in commercial information.^44^ Commercial sources may offer good quality information, especially if the topic is about invasive or risky interventions, as website providers may be more cautious.

Our study highlights a high prevalence of high trust in online health information, comparable with a study reporting that 64% of Americans have a high trust in online health information.^45^ However, other studies contrasted our findings, reporting lower levels of trust in online health information ranging between 34.8% and 23.9%.^46–48^ This tendency towards having high trust in online health information may be attributed to a lack of awareness or skills in evaluating the credibility of online health information, such as trusting online health information based on personal understanding and logical sense rather than using objective criteria.^14,49^

Our study revealed that 51.1% of the online health information was appropriately trusted by our participants. This finding is similar to a study by Pennycook et al.,^50^ which found that over 60% of responses accurately discerned true and false news headlines related to COVID-19 on social media. Although the eHealth literacy (mean eHEALS score: 29.2) and education level in our study are high, only 51.1% of the online health information entries were appropriately trusted. This suggests that evaluating the quality of online health information may depend on skills beyond educational and eHealth literacy levels.^51^ These skills include effectively using information and strategic Internet skills to find accurate information and make appropriate decisions based on that information.^52^

Our study revealed a higher appropriate trust in information sourced from websites compared to social media. This finding suggests that individuals struggle to discern the quality of health information on social media, even though they may recognise the presence of misinformation.^53^ This difficulty is likely due to the unmediated nature of social media platforms, which facilitate the unfiltered dissemination of unreliable, unverified, and contradictory information, thereby leading to uncertainty in trusting such information.^54^ The professional and structured nature of website content, along with the display of professional credentials and expertise, fosters a sense of trust among users, contrasting with the informal and heterogeneous nature of social media content.^55^ Older individuals may rely on simple heuristic cues rather than engaging in a cognitive effort to assess the quality of online health information.^56^ This reliance on heuristics may result in lower appropriate trust in social media, which tends to present information in a more simplified, graphical, and interactive format.^51^

Our study revealed a higher appropriate trust in non-commercial information compared to that with commercial intent. This finding is consistent with a study that revealed participants inappropriately trusted commercial-intended information despite knowing that the information is likely to be reused from different sources.^57^ Previous research has shown that individuals were more aware and proficient in evaluating the content of information from non-commercial sources.^58^ The strategic incorporation of misleading design and presentation in commercial information may pose a challenge for the public when determining its credibility.^59^ For instance, manipulation of information from credible sources out of context, citing outdated articles, or wrongly hyperlinking to scientific sources to deceive the public.^60^

Our study revealed a higher appropriate trust in information without misinformation compared to information with misinformation. When health misinformation is present, it can create confusion by downplaying risks, exaggerating benefits, and fostering misperceptions about products or conditions.^61–63^ This complicates efforts to assess the quality of information and may lead to uncertainty in trusting health sources. For instance, misinformation about statins has led to conflicting understanding compared to information provided by healthcare practitioners, contributing to patient distrust.^8^

Our study findings showed that the content focus of the information is not associated with appropriate trust in online health information quality. This finding suggests that specific content focus (whether the information is condition-related or general well-being) may not be a primary information characteristic when participants decide whether to trust online health information. This could be because participants often relied on their intuition and personal judgement to assess online health information content without objective standards.^14,64^

### Implications and recommendations

Our study showed that the high prevalence of poor-quality online health information underscores the necessity for health organisations and healthcare professionals to produce culturally adapted, high-quality information and guide patients towards these reliable sources. The results showed participants commonly exhibited inappropriate trust in online health information, underscoring the need for interventions aimed at improving their appraisal skills in online health information and learning to identify misinformation, as this may require specific skill sets. By understanding the information characteristics associated with the quality of online health information, policies can be developed to support the creation and dissemination of reliable health information. By identifying information characteristics associated with appropriate trust, we can better design interventions to educate patients in appraising online health information. The findings of this study will help develop trustworthy online information to assist patients in decision-making. Data captured using a real-time information diary tool provided nuance of how patients trust online information that is related to their health condition.

Future research could explore strategies to enhance online health information trustworthiness assessments and develop innovative tools to flag or alert internet users when encountering potentially poor-quality online health information or misinformation. Future research could explore how participants establish trust when encountering online health information, elucidating the factors that influence their trust and shedding light on the underlying mechanisms shaping patients’ perceptions of online health information credibility. This exploration could inform strategies to enhance trustworthiness assessments.

### Limitations

There were some limitations. The use of the DISCERN tool to assess information without therapeutic interventions can result in a lower score as there are seven questions in the tool related to treatment interventions. However, information without any therapeutic interventions was minimal in this study. Our study population exhibited a higher level of education and eHealth literacy, likely due to inclusion criteria requiring the use of a digital diary. Consequently, the findings of this study may not be generalisable to populations with lower education levels or eHealth literacy. Another limitation is that inter-rater reliability was assessed only for the presence of misinformation. We did not perform inter-rater reliability for other coding, such as the DISCERN score; however, both authors (WXL and Carmen) discussed each entry to reach a consensus.

## Conclusions

Our study found that patients with high cardiovascular risk exhibited appropriate trust in online health information primarily from websites, non-commercial information, and information free of misinformation. These findings suggest that evaluating online health information requires skills beyond traditional education and eHealth literacy. Therefore, there is a need to enhance patients’ skills in assessing the quality of online health information.

## Supplemental Material

sj-docx-1-dhj-10.1177_20552076251334438 - Supplemental material for Appropriate trust in online health information is associated with information platform, commercial status, and misinformation in patients with high cardiovascular risk Supplemental material, sj-docx-1-dhj-10.1177_20552076251334438 for Appropriate trust in online health information is associated with information platform, commercial status, and misinformation in patients with high cardiovascular risk by Woei Xian Lim, Hooi Min Lim, Yew Kong Lee, Carmen Jia Wen Chuah, Adina Abdullah, Chirk Jenn Ng and Adam G Dunn in DIGITAL HEALTH

sj-docx-2-dhj-10.1177_20552076251334438 - Supplemental material for Appropriate trust in online health information is associated with information platform, commercial status, and misinformation in patients with high cardiovascular risk Supplemental material, sj-docx-2-dhj-10.1177_20552076251334438 for Appropriate trust in online health information is associated with information platform, commercial status, and misinformation in patients with high cardiovascular risk by Woei Xian Lim, Hooi Min Lim, Yew Kong Lee, Carmen Jia Wen Chuah, Adina Abdullah, Chirk Jenn Ng and Adam G Dunn in DIGITAL HEALTH
