# Supplementary material for: Appropriate trust in online health information is associated with information platform, commercial status, and misinformation in patients with high cardiovascular risk
Source: Digit Health. 2025 Apr 29;11:20552076251334438. doi: 10.1177/20552076251334438 (PMC12048754; doi:10.1177/20552076251334438)
Supplement: sj-docx-1-dhj-10.1177_20552076251334438 - Supplemental material for Appropriate trust in online health information is associated with information platform, commercial status, and misinformation in patients with high cardiovascular risk [file sj-docx-1-dhj-10.1177_20552076251334438.docx]

| B1  A2  B2  A1 |  |
| --- | --- |
|  |  |

Supplementary 1: Distribution of the quality of information and level of trust across different information characteristics. (A1) Level of trust in platform; (A2) Quality of online health information in platform; (B1) Level of trust in commercial status (B2) Quality of online health information in commercial status; (C1) Level of trust in content focus; (C2) Quality of online health information in content focus; (D1) Level of trust in the presence of misinformation;(D2) Quality of online health information in the presence of misinformation

| C1 | C2 |
| --- | --- |
| D1 | D2 |
|  | |
