# Supplementary material for: Appropriate trust in online health information is associated with information platform, commercial status, and misinformation in patients with high cardiovascular risk
Source: Digit Health. 2025 Apr 29;11:20552076251334438. doi: 10.1177/20552076251334438 (PMC12048754; doi:10.1177/20552076251334438)
Supplement: sj-docx-2-dhj-10.1177_20552076251334438 - Supplemental material for Appropriate trust in online health information is associated with information platform, commercial status, and misinformation in patients with high cardiovascular risk [file sj-docx-2-dhj-10.1177_20552076251334438.docx]

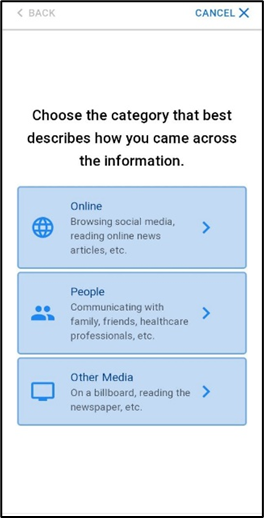

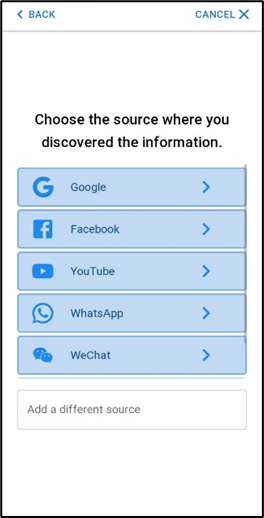

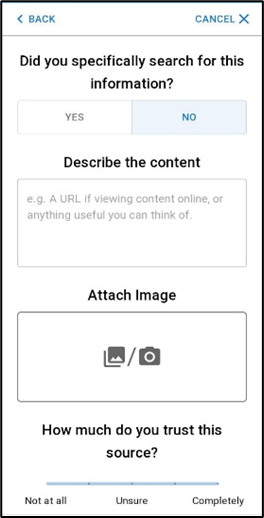


(C)

(B)

(A)

Supplementary 2: The user interface of the digital information diary tool. (A) Selecting the main source from either online, people or other media; (B) Choosing the sub-category such as the type of website or social media; (C) Determining their trust level based on a 5-point Likert scale.
